# Management of cell death in parasitic infections

**DOI:** 10.1007/s00281-021-00875-8

**Published:** 2021-07-19

**Authors:** Lidia Bosurgi, Carla V. Rothlin

**Affiliations:** 1 I. Department of Medicine, University Medical Center Hamburg-Eppendorf, Martinistrasse 52, 20251 Hamburg, Germany; 2 Protozoa Immunology, Bernhard Nocht Institute for Tropical Medicine, Bernhard Nocht Strasse 74, 20359 Hamburg, Germany; 3 Department of Immunobiology and Pharmacology, Yale University, New Haven, CT USA

**Keywords:** Parasites, Macrophages, Apoptosis, Apoptotic mimicry, Phosphatidylserine receptors

## Abstract

For a long time, host cell death during parasitic infection has been considered a reflection of tissue damage, and often associated with disease pathogenesis. However, during their evolution, protozoan and helminth parasites have developed strategies to interfere with cell death so as to spread and survive in the infected host, thereby ascribing a more intriguing role to infection-associated cell death. In this review, we examine the mechanisms used by intracellular and extracellular parasites to respectively inhibit or trigger programmed cell death. We further dissect the role of the prototypical “eat-me signal” phosphatidylserine (PtdSer) which, by being exposed on the cell surface of damaged host cells as well as on some viable parasites via a process of apoptotic mimicry, leads to their recognition and up-take by the neighboring phagocytes. Although barely dissected so far, the engagement of different PtdSer receptors on macrophages, by shaping the host immune response, affects the overall infection outcome in models of both protozoan and helminth infections. In this scenario, further understanding of the molecular and cellular regulation of the PtdSer exposing cell-macrophage interaction might allow the identification of new therapeutic targets for the management of parasitic infection.

## Introduction

For a long time, the death of the host cells during parasitic infections has exclusively been considered a manifestation of tissue damage, with critical consequences for disease pathogenesis. Many parasites have been initially characterized for their capacity to kill host cells. *Entamoeba histolytica* (*E. histolytica*), whose name reflects its ability to lyse host tissue, induces cell death via a caspase 3-like activity in a process which requires cell-to-cell contact [1] and in turn favors intestinal invasion. Similarly, upon infection with the *Plasmodium* parasite, induction of apoptosis, a form of programmed cell death, in endothelial cells reflects the blood-brain barrier disruption and critically contributes to the development of cerebral malaria [2].

However, it is now clear that host cell death, besides being a manifestation of tissue damage, is a tool the parasites take advantage of to survive within the host. In fact, the pathogens have evolved mechanisms to inhibit or induce cell death to create a beneficial environment which allows completion of the parasite life cycle. Promoting or preventing cell death becomes a beneficial or detrimental process for the pathogen and the host according to different variables intrinsic to the type of infection, such as the nature of the pathogen, the pathogen load, the site of infection as well as the time during the infection at which cell death occurs [3].

Upon infection with extracellular parasites, induction of cell death is most likely a pathogen strategy to reduce host fitness, allowing the pathogen to evade the immune response and thereby disseminate and persist into the host as known for instance in the case of infection with the protozoan *Trypanosoma brucei*, which is the driver of African trypanosomiasis, a disease mostly known as “sleeping sickness”. In contrast, intracellular parasites such as *Leishmania donovani* (*L. donovani*), a human blood protozoan responsible for visceral leishmaniasis, inhibits apoptosis of host macrophages in order to protect its niche for survival and replication [4].

In this review, we will emphasize both beneficial and detrimental aspects of parasite-regulated cell death on the host immune response. We will first highlight the mechanisms developed by different parasites to induce or prevent cell death, and secondly, once apoptotic cells are generated, how their sensing by professional phagocytes shapes the immune response and the consequent infection outcome.

## Parasite strategies to modulate host cell apoptosis

### Prevention of host cell death by protozoan parasites

Cell death is the primordial host defense strategy against intracellular infections: by inducing death of the infected cells, the host triggers death of the equivalent infecting pathogen. Therefore, intracellular pathogens can only ensure their survival and propagation by keeping the host cell alive, and hence have evolved different mechanisms to confer their resistance to apoptosis.

In 1994, the seminal work from Moore and Matlashewski first shed light on the capacity of parasites to modulate apoptotic cell death within their hosts. The authors described that infection of bone marrow-derived macrophages by *L. donovani* promastigotes led to secretion of mediators such as TNF-α and GM-CSF by the infected cells, with concomitant inhibition of the release of M-CSF and IL-1β. This altered cytokine environment in turn prevented apoptosis of the infected cells [5]. While this was the first of several studies describing the inhibition of host cell apoptosis by *Leishmania* strains, later elucidation of the mechanisms and the pathways involved in the parasite-host cell interaction led to the understanding that modulating cell death in the infected organism plays a fundamental role for parasite survival and, hence for the outcome of the infection.

Like other intracellular parasites, *Leishmania* relies on intact host cells to survive and propagate and have therefore evolved various strategies to subvert the host cell apoptotic machinery in order to guarantee their persistence within the infected host.

During the early phases of the infection, *Leishmania major* (*L. major*) induces delay of neutrophil apoptosis by around 24h via a mechanism involving the inhibition of caspase-3 activation. Due to the short lifetime of neutrophils, the parasite can only survive the first day intracellularly if their apoptosis is delayed, subsequently allowing these apoptotic neutrophils to be phagocytosed by macrophages which are recruited into the infected skin 1–2 days after infection. This delay, which is mediated by ERK1 and ERK2 activation [6], is essential to ensure that phagocytic macrophages “encounter” the apoptotic infected neutrophils, thus assuring the propagation of the parasite into the phagocytic macrophages upon their uptake [7]. Once inside the macrophages, the parasite has evolved several strategies which further help to preserve its niche within the host. For example, *L. donovani* inhibits growth factor deprivation-mediated apoptosis in macrophages via activation of the NF-kB and PI3K pathways [5, 8]. In addition, it prevents the oxidative burst-mediated apoptosis occurring in macrophages during the initial phases of the infection via reduction of caspase-3 and caspase-7 activation, and through the induction of suppressors of cytokine signaling (SOCS) proteins [9]. In line with this, SOCS1 and SOCS3 silencing in infected mice reduces parasite burden in both the liver and spleen. The protective effect of SOCS1 and SOCS3 inhibition has been associated with the alteration of the cross-talk between macrophages and T cells, which results in a switch from an anti-inflammatory to a pro-inflammatory cytokine environment characterized by the increasing levels of IL-12 and IFNγ, and the reduction in IL-10 and TGF-β synthesis. This in turn leads to decreased parasite survival [10]. To successfully survive within macrophages, *L. donovani* has also been described to delay host cell apoptosis via modulation of the unfolded stress response [11], and by inducing expression of MCL-1, an anti-apoptotic protein that, once translocated to the mitochondria, prevents cytochrome c release to inhibit mitochondrial dysfunction [12].

Not only *Leishmania* but also the sporozoite forms of the malaria parasite *Plasmodium* are able to inhibit host cell apoptosis in order to assure their propagation within the host. *Plasmodium* sporozoites, which constitute the first form of the malaria parasite to enter the human body, damage a number of hepatocytes during their migration through the liver. This damage leads to a liver stage, which is an obligatory gateway for parasite replication and a phase in the infection that for a long time has been considered clinically silent. Interestingly, during the liver stage, although the host cells possess the molecular machinery capable of sensing *Plasmodium* RNA, triggering a functional type I IFN response that reduces parasite burden [13], the *Plasmodium berghei*-infected hepatocytes showed increased resistance to apoptosis compared to the uninfected hepatocyte counterpart, and this prevents them from being attacked by the phagocytes [14]. Here, the active inhibition of host cell apoptosis via HGF/MET signaling through activation of the PI3K/Akt pathway is required for a successful infection [15]. Inside the hepatocytes, actually sporozoites appear to be surrounded by host LC3^+^ autophagy-derived vesicles, most likely used by the parasite as source of nutrients for growth [16]. Indeed, it is only after this stage that sporozoites will differentiate into merozoites and propagate inside the erythrocytes, a phase in the infection which is critical in triggering malaria-related pathology and all its complications [17].

Likewise, the capacity of the intracellular protozoan parasite *Cryptosporidium parvum* (*C. parvum*) to inhibit host cell death has also been investigated. *C. parvum* is distributed worldwide and is the leading cause of diarrhea morbidity and mortality in children younger than 5 years of age. It is generally transmitted from person to person via the fecal-oral route, mainly through contaminated water supplies. Infection in vitro of a human tumor cell line helped clarify that *C. parvum* replication occurs only in the infected cells in which apoptosis is inhibited [18]. Inhibition of apoptosis in the infected cells arises through activation of NF-kB [19] and consequent upregulation of inhibitors of apoptosis proteins (IAPs) genes including c-IAP1, c-IAP2, and XIAP, which are known to specifically prevent TNF-induced cell death [20], and surviving [21]. A similar mechanism of inhibition of apoptosis which involves transcription of NF-kB-dependent genes also occurs upon infection with a different intracellular parasite, the protozoan T*oxoplasma gondii* (*T. gondii*). *T. gondii* infection is widespread in humans, with most of the infected people being asymptomatic or showing mild symptoms. However, especially when the infection occurs congenitally via infection of a pregnant woman, it can cause severe diseases often associated with ocular disorders in the newborn [22]. The parasite is able to infect almost all nucleated cells in the host. In vitro experiments in which a fibroblast cell line is infected with *T. gondii* suggest that induction of transcription of NF-kB-dependent genes with antiapoptotic properties (such the two Bcl-2 members *Bfl-1* and *Bcl-x*_*L*_) in the infected cells is one of the mechanisms the parasite uses to inhibit host cell apoptosis [23]. Along the same line, increase in the expression of the microRNA miR-17-92 in *T. gondii*-infected macrophages triggers the downregulation of the pro-apoptotic Bcl-2-family gene *Bimp1* [24]; therefore, pointing out that manipulation of the balance between pro- and anti-apoptotic Bcl-2 family members as well as the inhibition of caspase-9, caspase-3, and JNK activation, all leads to blocking of apoptosis during *T. gondii* infection [25].

In addition to this, in an early report, cells infected with *T. gondii* have been described to be resistant to apoptosis initiated by multiple triggers, such as Fas-dependent and Fas-independent cytotoxic T lymphocytes-mediated cytotoxicity, IL-2 deprivation, gamma irradiation, UV irradiation, and the calcium ionophore Beauvericin [26]. Here, inhibition of apoptosis has been linked to different mechanisms in which the presence of a viable parasite is critical, as confirmed by the reversal of apoptosis resistance upon killing of intracellular parasites with antibiotics [26]. Viable parasites prevent apoptosis in host cells via the inhibition of cytochrome c-induced caspase activation, and in particular, through the release of molecules which interferes with cytochrome *c*/dATP-induced activation of the caspase cascade [27] or directly degrading the initiator caspase-8 [28].

Interestingly, during infection with *T. gondii*, not only inhibition of host cell death but also its induction has been hypothesized. Mice inoculated with a highly virulent *T. gondii* strain (RH) uniformly succumbed to infection, compared to mice infected with a low-virulence strain. In the spleen of these RH/high virulence strain-infected mice, numerous clusters of apoptotic cells were detected by TUNEL at day 8 post-infection. In this scenario, the increased amount of dying cells was associated with the collapse of the splenic architecture and the detection of high levels of IFN-γ, an inflammatory cytokine that, when over-secreted, can lead to pathology and death [29]. However, whether the induction of apoptosis represented an active strategy the parasite used to evade the immune response, rather than the consequence of the high levels of activation of splenic cells in response to infection, was not clear [30]. To address this point, analysis of pathology in organs other than the lymphoid tissue, such as the liver, led to the understanding that host cell apoptosis was not the consequence of injury, as reflected by the fact that very few TUNEL-positive hepatocytes were detected in the damaged liver. In addition, as previously mentioned, extensive apoptosis was found in the spleen of the infected mice but with cell death not occurring in infected cells, but rather in T cells and NK cells which localize at sites distal from parasite replication [31]. These data therefore suggest that in the context of *T. gondii* infection, the parasite might still protect the infected cell from apoptosis*,* while cell death is efficiently induced in non-infected cells.

### Induction of host cell death by protozoan parasites

Under certain circumstances, some protozoan parasites can induce apoptosis of the infected cells, rather than inhibiting it, so that the consequent uptake of the infected cells by neighboring healthy ones assures the propagation and dissemination of the parasite within the host. For instance, *E. histolyca*, upon cell contact with human neutrophils, induces their apoptosis in a process which is characterized by NADPH oxidase-derived ROS production and ERK1/2 activation [32]. This induced host cell death contributes to disease pathogenesis [32, 33], as observed in infected mice in which treatment with the pan-caspase inhibitor zVAD-fmk leads to the reduction of the liver abscess size by 70% compared to the control group [34].

The impact of the infection with the obligate intracellular parasite *Trypanosoma cruzi* (*T. cruzi*) on host cell death and in particular on the apoptosis of T lymphocytes has also been extensively studied. Infection with *T. cruzi* causes “Chagas” disease, affecting around 7 million people worldwide, and which can occur in both an acute and chronic form leading to major pathologies including damage to the heart and digestive tract. The gastrointestinal tract, particularly the colon and/or stomach, represents a permissive niche for the parasite, with increased in disease severity being correlated with the spreading of the parasite to the cardiac muscle [35].

Apoptosis of T lymphocytes during the contraction phase of an immune response often occurs through a process defined as activation-induced cell death, caused by re-stimulation of already activated T cells [36]. This process is usually triggered by expression of death receptors on the T cells, such as Fas Receptor (Fas), whose engagement via Fas Ligand (FasL) leads to caspase activation [37]. Apoptotic CD4 T cells have been detected in spleen and lymph nodes during the acute stage of *T. cruzi* infection [38, 39], but also in the heart of chronically infected patients [40]. Both CD4 and CD8 T cells start to express Fas during the course of the infection, mirroring an activation-induced cell death [41]. Similarly, increased levels of FasL have been detected in the serum of patients chronically infected with *T. cruzi* [42]. That apoptosis can be considered the cause rather than the consequence of disease severity in this context has been proved in a murine model of *T. cruzi* infection, in which a single injection of apoptotic but not necrotic or vital T cells in infected mice increased their parasitemia [43]. Conversely, reduction of host cell apoptosis via treatment with the caspase inhibitor zVAD decreases the number of parasites through induction of memory/effector CD8 T cells in the infected tissue and increases the number of pro-inflammatory macrophages with high capacity to secrete IL-12 [44]. These experiments have been correlated to a series of in vitro analyses in which coculture of apoptotic T cells with peritoneal macrophages was shown to predispose macrophages to infection with *T. cruzi*. Again, the exacerbation in parasite growth was inhibited when macrophages were pre-exposed to T cells in which apoptosis was blocked via treatment with the pan-caspase inhibitor zVAD-fmk [43] or when macrophages were cocultured with CD8 T cells in the presence of anti-FasL [45]. These data are in line with the observation that a suboptimal CD8 T cell response in the context of *T. cruzi* is associated with expression of Fas and the acquisition of a pro-apoptotic phenotype. In this scenario, immunization with an adenoviral vaccine expressing a parasite immunodominant antigen leads to expansion of CD8 T cells with impaired capacity to express Fas and thus undergoing into apoptosis, which therefore leads to protective immunity [41].

### Helminth strategies to induce host cell death

In contrast to many of the intracellular parasites reported here, extracellular pathogens do not rely on the integrity of defined host cells for survival, unless this affects host viability. In this case, the parasite often induces apoptosis in host cells to impair their anti-pathogen response and thus guarantee the parasite survival and propagation into the host. Induction of apoptosis of the cells of the innate and adaptive immune system might indeed represent the most efficient tool to dampen the host immune response against the pathogen. In light of this, the mechanism leading to apoptosis of immune cells, such as for instance apoptosis of T cells during the infection with the helminth *Schistosoma*, has been examined by several groups.

Schistosomiasis is a chronic debilitating disease that currently affects 200 million people worldwide and is characterized by an initial Th1 response that later evolves to a parasite antigen-driven Th2 response. Among the various factors that have been considered as drivers of the Th1-Th2 switch, apoptosis of T cells during the course of the infection also seems to contribute [46]. Interestingly, by comparing the immunopathology induced upon infection in two different strains of mice which develop high or low pathology, as mirrored by the size of granuloma developed in the liver, it was observed that T cell apoptosis is significantly higher in *Schistosoma*-infected mice of the strain that develops low pathology. This proves that in this model of helminth infection the magnitude of CD4 T cell-mediated immunopathology correlates inversely with the extent of CD4 T cell apoptosis. In addition, within the granuloma, which are organized aggregates of immune cells surrounding the parasite egg, apoptosis of T cells was linked to detection of lower amounts of IL-2, suggesting growth factor deprivation as the main cause of cell death [47].

However, not only IL-2 withdrawal but also antigens from the *Schistosoma* parasites have been described to trigger apoptosis in vitro, as observed when T cells isolated from the blood of chronically infected patients were cocultured with the soluble egg antigen (SEA) from *Schistosoma mansoni* (*S. mansoni*) [48]. SEA from *Schistosoma japonicum* has also been described to induce apoptosis in hepatic stellate cells (HSC) via activation of TRAIL/DR5 death receptor-dependent apoptosis occurring through a caspase-dependent mechanism [49]. This in turn leads to suppression of HSC activation through PPARγ and the TGF-β signaling pathways, and therefore suggests a potential role for the SEA in attenuating liver fibrosis [49]. In general, proteins that are actively secreted by the parasite or are the result of passive leakage from its surface are named excretory-secretory products (ESP) and play a key role in mediating host cell apoptosis during infection. Identifying the components of SEA and of ESP in general is of special interest, considering for instance that in in vivo experiments, ESP released from skin stage schistosomula of *S. mansoni* are described to induce expression of Fas, FasL, and FADD in skin draining lymph node T cells [50].

Interestingly, CD4 T cells isolated from mice infected with *S. mansoni* multiple times showed the propensity to become anergic, most likely because of the increased capacity to undergo apoptosis as detected by increased levels of Fas and FasL [51]. The induction of apoptosis in CD4 T cells was mediated by IL-10, since in IL10^−/−^ mice and upon neutralization of IL-10 in vitro, CD4 T cell activation was restored and apoptosis reduced [46, 51]. However, while apoptosis in T cells has frequently been reported, with granuloma lymphocytes being more susceptible to Fas-FasL-mediated apoptosis than spleen lymphocytes, eosinophils seem to be resistant to apoptosis, although they express significant levels of Fas [52]. What are the critical triggers that drive apoptosis only in selective cell types and what are the consequences of this process in schistosomiasis have not been described so far.

During infection with a different trematode, the helminth *Fasciola hepatica*, apoptosis of the host immune cells has also been reported. Here, ESPs trigger apoptosis in macrophages reaching the infected peritoneum [53] as well as in eosinophils, in a process regulated by tyrosine kinases and activation of caspase-3, caspase-8, and caspase-9 [54]. In particular, sensing of the parasite ESP by eosinophils triggers their cell death through an increase in ROS production, mainly H_2_O_2_, which is then followed by a process of mitochondrial membrane depolarization that precedes caspase activation [55]. ESPs from the nematode *Onchocerca volvulus* are also able to induce cell death in vitro. Here, late apoptosis detected via the increase in the frequency of Ann-V^+^7-AAD^+^ cells was induced in splenocytes exposed to OvALT-2 and OvNLT-1, two *Onchocerca volvulus*-derived proteins [56]. In addition to immune cell death, stromal cell apoptosis also occurs during helminth infection. TNF-α and IFNγ secreted in response to the chronic damage caused by the gastrointestinal parasite *Trichuris muris* trigger epithelial cells apoptosis in the large intestine of infected mice. Here, the host immune response rather than the physical damage caused by the parasite appears to be responsible for the increase in host cell death [57].

## Phosphatidylserine exposure on the infecting parasite: A strategy for initiation and maintenance of infections

Phosphatidylserine (PtdSer) is a phospholipid normally present in the inner leaf of the lipid bilayer of living cells, thanks to the activity of ATP-dependent phospholipid flippases. When cells undergo apoptosis, loss of flippase activity and activation of scramblase leads to PtdSer shifts on the outer leaflet of the cell membrane, serving as “eat-me signal” for the neighboring phagocytes [58]. Recognition of PtdSer via receptors expressed in phagocytes guarantees the removal of the dying cells via phagocytosis [59]. Hence, while PtdSer exposure on the cell surface of apoptotic cells might simply be considered a tag used to be recognized by the surrounding phagocytes, exposure of PtdSer on the membrane of viable intracellular parasites represents a strategy the pathogens use to efficiently infect the surrounding phagocytes, thus guarantying parasite maintenance in the host. This process is defined as “classical” apoptotic mimicry and has been observed, for instance, in the context of *Leishmania* infection, where PtdSer-exposing amastigotes can be purified from the mouse lesions. The amastigotes, once recognized by the phagocytic macrophages, makes the phagocytic cells more susceptible to parasite growth by altering their activation status and inducing the secretion of IL-10 and TGF-β while reducing NO production [60]. Interestingly, the exposure of PtdSer on viable amastigotes appears to be dependent on low concentration of NO present in the environment [61].

Although not considered part of the process of apoptotic mimicry, other mechanisms involving the exposure of PtdSer have been developed by the pathogens to guarantee the perpetuation of the infection. For instance, a population of PtdSer^pos^ *Leishmania* promastigotes has been described during infection. This subpopulation, although being apoptotic and meant to die, is critical in determining virulence by providing survival advantage for the non-apoptotic parasites (PtdSer^neg^) within the infected neutrophils. This process, which is mediated by TGF-β, results in disease development [62]. Therefore, in the context of *Leishmania* infection, exposure of PtdSer on a viable amastigote or on the dying proamastigotes, via different strategies, contributes to disease progression.

An apoptotic phenotype has also been described in the case of *T. cruzi*. *T. cruzi* undergoes three different developmental stages: replicative epimastigotes found in the vector, intracellular replicative amastigotes and trypomastigotes. During the transition from the proliferative to the stationary growth phase in vitro, *T. cruzi* epimastigotes expose PtdSer, undergo nuclear DNA fragmentation, and increase caspase-3-like activity [63]. In a different experimental setting, *T. cruzi* trypomastigotes, the non-replicative form of the parasite that initiates the infection in vertebrate hosts and thus interact with the host innate immune cells, have also been shown to expose PtdSer on their surface. Interestingly, upon coculture of the trypomastigotes with macrophages in vitro, the exposure of PtdSer correlates with triggering of the TGF-β pathway and reduction in iNOS expression in the infected macrophages, thus suggesting sabotaging of the macrophage microbicidal system by the parasite [64].

Likewise, exposure of PtdSer also occurs in a fraction of the *T. gondii* parasite population during infection. Contrarily to other infection models, here, only the PtdSer^neg^ parasites were taken up by macrophages. Conversely, the PtdSer^pos^ parasite fraction invaded the macrophages by active penetration. Interestingly, the total *T. gondii* population, which was a combination of both the PtdSer^neg^ and PtdSer^pos^ parasite fractions, was necessary for the successful infection of macrophages cultured with the parasite in vitro. The two fractions exerted different functions, with the PtdSer^pos^ subpopulation of *T. gondii* being the only one able to inhibit production of microbicidal molecules such as NO by activated macrophages. In contrast, most likely due to the inability to block NO, the PtdSer^neg^ parasite fraction led to a stronger inflammatory response in vivo, as reflected by the high number of macrophages detected in the infected tissue compared to mice infected with PtdSer^pos^ parasite [65].

## The role of macrophages in the clearance of dying cells during parasitic infection

During an infection, efficient removal of pathogens or swift clearance of dying cells needs to occur promptly. This process not only represents an efficient defense strategy against the invading parasite, but is also critical to clear dead host cells and thereby assure the re-establishment of tissue homeostasis. However, as reported above, in some circumstances, infected apoptotic cells, once taken up by surrounding phagocytes, serve as Trojan Horses for effective dispersion and survival of the parasite within the host. In the next section, we will discuss the effects of the clearance of either infected or uninfected PtdSer-exposing cells on phagocytic macrophages and how this impacts the course of the infection.

### PtdSer receptors in phagocytic macrophages

Expression of various phagocytic receptors, with different structures and efficiency to bind high levels of PtdSer, has been detected on professional phagocytes such as macrophages. While single-cell sequencing analysis might reveal specificity between expression of certain phagocytic receptors and selective subsets of macrophages, the fact that macrophages often concomitantly express more than one phagocytic receptor at a time underlines their importance for the proper removal of dying cells. Functional redundancy among the at least 12 described PtdSer phagocytic receptors exists, with some of them recognizing and taking up apoptotic cells via direct binding to PtdSer, and some others using bridging molecules as sort of co-receptors for PtdSer recognition.

**Fig. 1:**
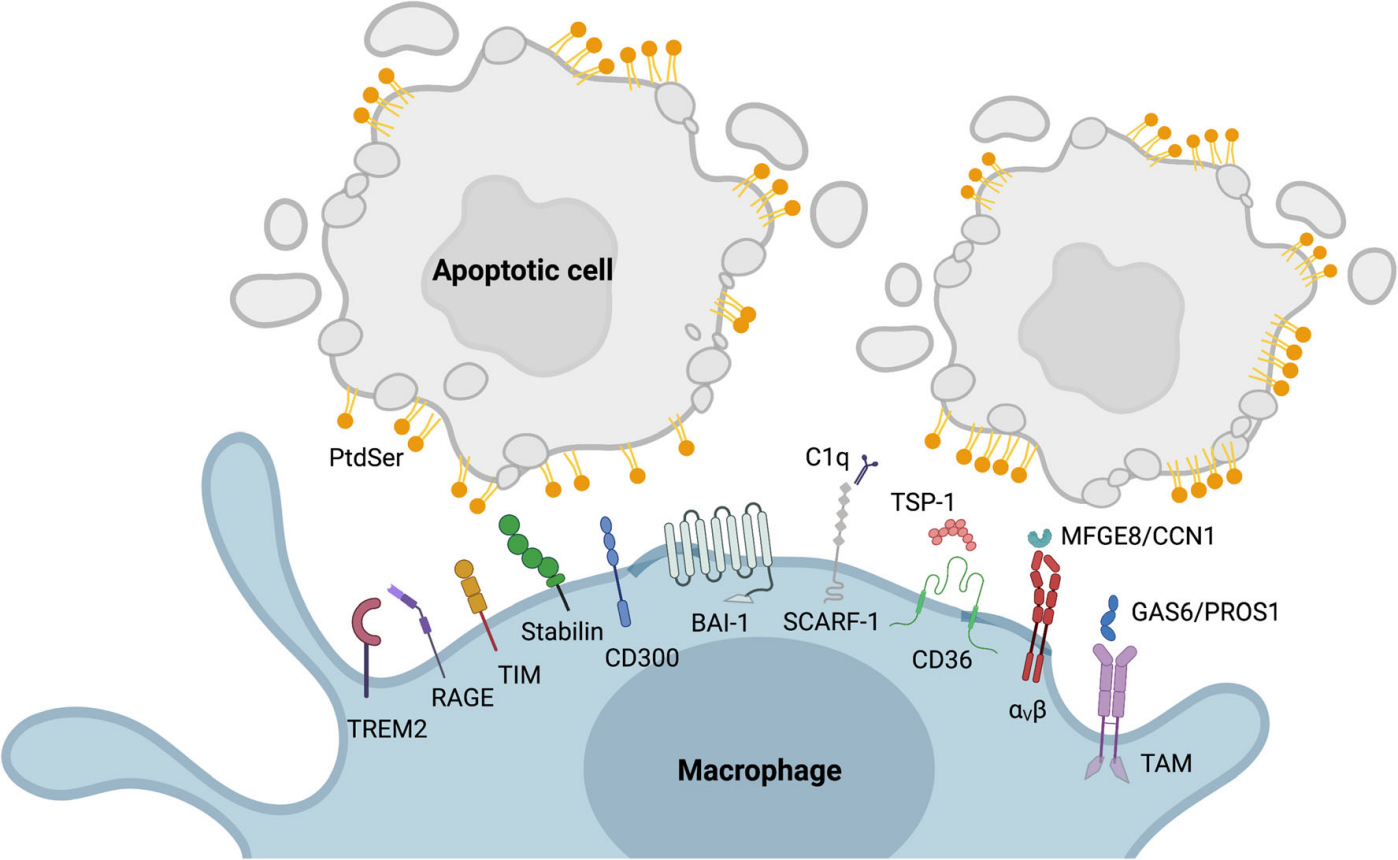
PtdSer receptors and bridging ligands mediating the recognition and uptake of apoptotic cells by macrophages. The “eat-me” signal PtdSer is exposed on the outer leaflet of the plasma membrane of cells undergoing apoptosis. Recognition and uptake of apoptotic cells occurs in macrophages via the engagement of a series of different PtdSer receptors, which bind PtdSer either directly or indirectly through bridging ligands. Examples of PtdSer receptors and corresponding bridging ligands are reported

Stabilin receptors (STAB1 and STAB2), T cell immunoglobulin and mucin domain-containing molecule TIM (TIM1, TIM3, TIM4), brain angiogenesis inhibitor 1 (BAI-1), CD300 (CD300A, CD300B, CD300F), receptor for advanced glycation end products (RAGE), and triggering receptor expressed by myeloid cells-2 (TREM-2) are example of receptors which directly bind the PtdSer exposed on apoptotic cells. In contrast, AXL and MERTK belong to a family of receptor tyrosine kinases named TAM (TYRO 3, AXL, and MERTK), which bind PtdSer via their cognate ligands GAS6 and PROS1 and exert a critical role in the resolution of inflammation through the up-take of apoptotic cells [66]. The integrins α_v_β5 or α_v_β3 and also the scavenger receptors SCARF-1 and CD36 use different bridging molecules such as, respectively, MFGE8 and CCN1 and C1q and TSP-1 for PtdSer recognition and signaling (Fig. 1 and reviewed in [67, 68]). Some of these receptors have been proposed to act in synergy with other receptors/efferocytosis molecules for the efficient uptake of apoptotic cells. Integrins, for instance, together with the glycoproteins CD36, expressed by the phagocytic cells, and TSP-1, expressed on apoptotic cells, create a phagocytically active complex which allows the uptake of the apoptotic cells. Synergy between αvβ5 and MERTK or STAB2 as well as cooperation between TIM-4 and MERTK has also been reported [69–71].

### The impact of PtdSer receptor engagement on parasitic infections

While the role of PtdSer receptors has been extensively studied in the context of inflammation induced by sterile damage, such as autoimmune disease or cancer, very little is known on the identity of PtdSer phagocytic receptors expressed by macrophages as well as the impact of phagocytosis of PtdSer^pos^ cells during parasitic infections.

Several data have been generated in the context of malaria disease, in which phagocytosis of *Plasmodium falciparum*-infected erythrocytes is mediated by macrophages expressing the receptor CD36 [72]. CD36 is one of the best-studied class B scavenger receptors, which is capable of eliciting a variety of signaling responses according to the ligand it interacts with [73]. Early studies identified interaction between CD36 and PtdSer-containing membranes [74], and this was in line with the results showing that *Plasmodium*-infected red blood cells suffer great stress and expose PtdSer [75]. In vitro CD36 expression can be induced via treatment with PPAR-γ/RXR agonists, which in turn increases macrophage phagocytosis of infected erythrocytes [76]. During this process, CD36 clustering leads to activation of both ERK and p38MAPK signaling cascades [72]. Consistent with this, CD14^+^CD16^-^ and CD14^+^CD16^hi^ blood monocytes from children with acute malaria show impaired expression of CD36 and reduced phagocytic capacity in vitro compared to samples isolated from children six weeks after recovery [77], therefore suggesting that lower CD36 expression by two population of monocytes (CD14^+^CD16^−^ and CD14^+^CD16^+^) is related to severity of the infection and consequent death [78]. These data are strongly supported by the fact that African populations contain an exceptionally high frequency of mutations in CD36, which, by causing CD36 deficiency, are associated with susceptibility to severe malaria [79].

Interestingly, while on the one hand, phagocytosis of infected erythrocytes might be beneficial to eliminate the pathogen, on the other hand, it is associated with development of anemia that is one of the major clinical hallmarks of malaria in patients, and whose pathophysiology is not yet completely understood. The use of a model of infection with *Plasmodium yoelii* (*P. yoelii*), which has the capacity to induce apoptosis in both infected and non-infected host cell [80] and induce strong anemia in mice, allowed the identification of antibodies which recognize PtdSer exposed on the surface of non-infected erythrocytes [81]. These antibodies, by mediating the clearance of the uninfected erythrocytes during the course of the disease, lead to strong anemia, which can be reversed when mice receive the PtdSer-binding protein Annexin-V [81].

In parallel, phagocytosis of *P. yoelii*-infected erythrocytes also occurs during the course of the disease and is mediated by the receptor TIM-4, as observed in vitro, where treatment with anti-TIM-4 antibody blocks the uptake of parasitized red blood cells by macrophages in a dose-dependent manner [82]. The phagocytic macrophages mentioned here are a population of F4/80^+^ red pulp cells which do not express SIGNR1, CD169, nor CD68, characteristic markers of marginal zone, marginal metallophilic, and tingible body macrophages, respectively. However, they express TIM-4, receptor which cannot complete signal transduction by itself, due to the absence of a defined signaling domain [83]. Likewise, TIM-4 does not support efferocytosis by itself, but utilizes integrins as co-receptors [84] and enhances phagocytosis mediated by the family of receptor tyrosine kinases TAM [85]. Based on this, although TIM-4 may play a major role, other PtdSer receptors might also be involved in the uptake of infected cells in malaria.

Among the various PtdSer phagocytic receptors, the role of CD300F in parasitic diseases has also been investigated, although to the best of our knowledge, only in a transgenic mouse model resistant to the development of cerebral malaria upon infection with *P. berghei ANKA.* CD300F belongs to a family of type I transmembrane proteins which binds PtdSer, thus enhancing the phagocytosis of apoptotic cells [86]. Upon infection, CD11b^+^CD45^hi^Ly6C^hi/int^ brain macrophages sorted from cerebral malaria-resistant mice show higher expression levels of CD300F compared to microglia isolated from mice which developed cerebral malaria. Here, CD300F plays a role in dampening the inflammatory response during infection, most likely via reduction of CXCL10, TNF-α, and IFN-γ production in microglial cells [87].

Different receptors which mediate PtdSer-dependent phagocytosis have been described in the internalization of *Leishmania* by macrophages. Indeed, treatment with the PtdSer-binding protein Annexin-V was used to mask PtdSer at the surface of *Leishmania* amastigotes and reduce the parasite uptake by macrophages [88]. Under steady-state conditions, a population of dermal macrophages expressing high levels of the mannose receptor and characterized by high capacity to phagocyte apoptotic cells, as mirrored by expression of the PtdSer phagocytic receptors MERTK and TIM-4, is the primary target of *L. major* infection. These macrophages, whose anti-inflammatory function is maintained during infection via IL-4 and IL-10, through a CCL24-mediated amplification loop [89], represent a permissive niche for parasite growth and might be a target for the treatment of the chronic form of cutaneous leishmaniasis [90]. Interestingly, neutrophils recruited at the site of infection, which function as Trojan Horses vectoring *L. major* into macrophages, are taken up by dermal macrophages in a process which is AXL and MERTK dependent and that controls macrophage anti-inflammatory function as well as severity of the lesion and parasite burden [91].

Besides their role during the course of infection with the intracellular parasite *L. major*, AXL and MERTK expression has been also detected during the inflammatory response induced by the helminth parasite *Nippostrongylus brasiliensis* (*N. brasiliensis*) [92]. The nematode *N. brasiliensis*, a rat hookworm that also causes natural infections in mice, is a gastrointestinal parasite and close relative of human hookworms. Infection with the nematode results in a Type-2-dominated immune response. Damage initially occurs in the lung, where accumulation of apoptotic neutrophils arises. Here, AXL and MERTK expressing lung macrophages acquire a tissue remodeling function by efficiently clearing dying neutrophils. In contrast, *Axl*^*−/−*^*Mertk*^*−/−*^ macrophages isolated from the infected lung show reduced levels of anti-inflammatory and tissue repair genes as *Retnla* and *Alox15*. Interestingly, AXL and MERTK expression seems to be dispensable for controlling lung tissue damage during the initial stage of the infection, as mirrored by the similar amount of red blood cells detected in the bronchoalveolar lavage of WT and *Axl*^*−/−*^*Mertk*^*−/−*^ mice [92]. Very recently, expression of the three TAM receptors as well as their ligand GAS6 has been described to be differentially modulated in circulating leukocytes from multiple sclerosis (MS) patients chronically infected with helminths, compared to uninfected MS patients. While AXL and MERTK expression was increased in circulating DCs, monocytes showed increased levels of GAS6 and PROS1 in infected MS patients compared to non-infected ones. Modulation of the TAM axis by helminths in turn tempers the innate immune response and reduces the development of a pathogenic Th17 response in MS patients [93].

A role for the integrin α_v_β3, encoded by the gene *Itgb3*, has been described in the context of *T. cruzi* infection, where macrophages use it to uptake apoptotic cells. This event, by leading to TGF-β secretion, renders macrophages unable to exert NO-dependent trypanocidal activity and in turn favors parasite replication [43]. A role for the integrin α_v_β3 has also been extensively described in the context of malaria infection. Here, its function is not directly related to PtdSer binding, but instead critical for cytoadhesion of parasite-infected erythrocytes to the endothelium [94] [95]. Very recently, α_v_β3 has also been identified as a receptor for the *P. falciparum* surface ligand TRAP, a typical type I cell surface protein containing both a von Willebrand factor A and a thrombospondin type 1 repeat domain. TRAP is necessary for parasite motility, as observed in *Itgb3*-deficient mice, in which sporozoites were able to move faster within the dermis, therefore suggesting that the TRAP-α_v_β3 interaction could impede the movement of the parasite [96].

**Fig. 2:**
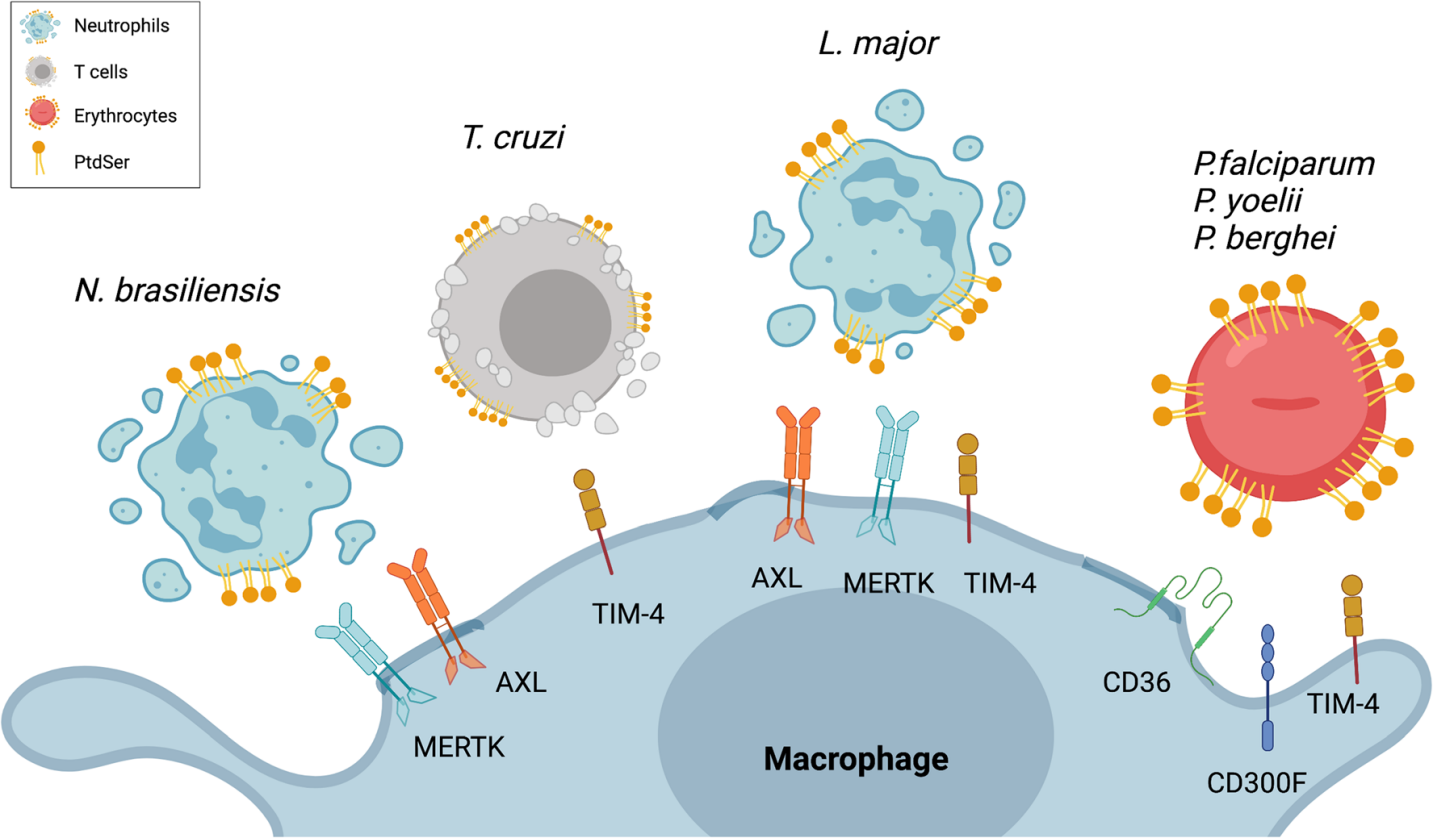
Engagement of PtdSer receptors and consequent uptake of apoptotic cells by macrophages regulates the course of parasitic infections. According to the type of parasite infecting the host, either infected or uninfected apoptotic cells with different identities (e.g., T cells, neutrophils, erythrocytes) are generated. PtdSer exposed on their cell membrane is recognized by a variety of different PtdSer receptors expressed by macrophages. This interaction, by regulating the clearance of apoptotic cells by macrophages, is an important regulator of the host response during infection

While characterization of PtdSer^pos^ and PtdSer^neg^ parasites has been performed in the context of infection with the obligate intracellular parasitic *T. gondii*, and anti-PtdSer antibodies are frequently detected during infection, the role of PtdSer receptors on phagocytic cells has not been analyzed so far. Infection with the PtdSer^pos^ subpopulation of *T. gondii* inhibits NO production by activated macrophages [65], an effect that in vitro has been described to be caused by the engagement of the PtdSer receptor TIM-4 in the context of LPS and IFNγ stimulation [97]. A schematic summary of the phagocytic receptors engaged by macrophages for the uptake of infected or uninfected apoptotic cells during different parasitic infections is reported in Fig. 2.

## Conclusions

In this review, we have discussed various aspects regarding the modulation of host cell death by parasites and the consequent handling of dying cells by phagocytic macrophages. Several of these are the strategies the parasite has developed to turn host cell death to its own advantage or to acquire an apoptotic-like phenotype to more easily propagate within the host. Furthermore, we have highlighted some of the latest discoveries on the mechanisms of sensing infected and non-infected apoptotic cells by professional phagocytes, with particular attention to the engaged PtdSer-receptors and their overall impact on the course of the infection.

Recently, a series of preclinical evidence highlighting the role of phagocytosis mediated by macrophages has been in the spotlight, as observed via the use of phagocytosis checkpoint inhibitors in cancer treatment [98] as well as the generation of Chimeric Antigen Receptors for Phagocytosis (CAR-P) [99], which direct macrophages to specifically engulf cancer cells. Although targeting phagocytosis and its mediators might indeed contribute to the development of novel therapeutic tools for various inflammatory diseases, still very little has been done so far to dissect the principles at the basis of the phagocytic process and its therapeutic potential in the context of parasitic infections.

Few studies have reported the beneficial effect of macrophage-based cell therapy in parasitic diseases, for example, in the context of infection with helminth parasites *N. brasiliensis* or *S. mansoni*. Here, the advantageous effect of macrophage transfer relied on the acquisition of a specific macrophage activation status rather than on their phagocytic capacity. Transfer of macrophages leads indeed to host protection against a lethal *N. brasiliensis-*induced inflammation, thanks to their capacity to respond to type 2 cytokines acquiring a tissue remodeling profile [100]. Likewise, transferring bone marrow-derived monocytes in a model of chronic schistosomiasis leads to reduction of liver fibrosis, and among the various factors analyzed is the decrease in Galectin 3 expression which is important for activation of macrophages with a pro-fibrotic profile [101]. However, respective data on the phagocytic capacity of the transferred macrophage and how this might impact the infection outcome are scarce.

Many of the strategies reported in this review are used by parasites which cause diseases classified as Neglected Tropical Diseases (NTDs), such as schistosomiasis, Chagas disease, and leishmaniases, for which novel or improved treatments are urgently needed. Blocking macrophage capacity to uptake infected apoptotic cells might represent an alternative approach to inhibit the survival and propagation of many intracellular parasites. However, blockage of phagocytic receptors on macrophages via, for instance, systemic administration of phagocytic receptor small molecule inhibitors will lead to reduced engulfment of most of the apoptotic cells generated, independent of their infection status. This in turn might limit the macrophage tissue remodeling response naturally resulting from the uptake of non-infected apoptotic cells. Similarly, blocking phagocytosis during helminth infections might suppress the generation of an anti-inflammatory response and therefore prevent tissue regeneration.

These downsides hint toward the development of therapeutic strategies aiming at manipulating phagocytosis to promote only the engulfment of selective targets. The use, for instance, of CAR-P technology might in the future unleash the power of phagocytosis, leading to the generation of an immune environment in which macrophages are reprogrammed to taking up only uninfected damaged cells, thus avoiding the detrimental consequences of taking up cells infected, for instance, with intracellular protozoan.

In conclusion, understanding the mechanisms behind phagocytosis regulation and further characterizing the molecules driving the macrophage/apoptotic cell cross-talk may open up new venues for the treatment of various inflammatory disease and alternative approaches for the management of parasitic infection and in particular NTDs.
